# Fat Oxidation, Fitness and Skeletal Muscle Expression of Oxidative/Lipid Metabolism Genes in South Asians: Implications for Insulin Resistance?

**DOI:** 10.1371/journal.pone.0014197

**Published:** 2010-12-01

**Authors:** Lesley M. L. Hall, Colin N. Moran, Gillian R. Milne, John Wilson, Niall G. MacFarlane, Nita G. Forouhi, Narayanan Hariharan, Ian P. Salt, Naveed Sattar, Jason M. R. Gill

**Affiliations:** 1 Institute of Cardiovascular and Medical Sciences, University of Glasgow, Glasgow, United Kingdom; 2 School of Life Sciences, University of Glasgow, Glasgow, United Kingdom; 3 MRC Epidemiology Unit, Institute of Metabolic Science, Addenbrooke's Hospital, Cambridge, United Kingdom; 4 Pfizer Global Research and Development, Collegeville, Pennsylvania, United States of America; University of Las Palmas de Gran Canaria, Spain

## Abstract

**Background:**

South Asians are more insulin resistant than Europeans, which cannot be fully explained by differences in adiposity. We investigated whether differences in oxidative capacity and capacity for fatty acid utilisation in South Asians might contribute, using a range of whole-body and skeletal muscle measures.

**Methodology/Principal Findings:**

Twenty men of South Asian ethnic origin and 20 age and BMI-matched men of white European descent underwent exercise and metabolic testing and provided a muscle biopsy to determine expression of oxidative and lipid metabolism genes and of insulin signalling proteins. In analyses adjusted for age, BMI, fat mass and physical activity, South Asians, compared to Europeans, exhibited; reduced insulin sensitivity by 26% (p = 0.010); lower VO_2max_ (40.6±6.6 *vs* 52.4±5.7 ml.kg^−1^.min^−1^, p = 0.001); and reduced fat oxidation during submaximal exercise at the same relative (3.77±2.02 *vs* 6.55±2.60 mg.kg^−1^.min^−1^ at 55% VO_2max_, p = 0.013), and absolute (3.46±2.20 *vs* 6.00±1.93 mg.kg^−1^.min^−1^ at 25 ml O_2_.kg^−1^.min^−1^, p = 0.021), exercise intensities. South Asians exhibited significantly higher skeletal muscle gene expression of CPT1A and FASN and significantly lower skeletal muscle protein expression of PI3K and PKB Ser473 phosphorylation. Fat oxidation during submaximal exercise and VO_2max_ both correlated significantly with insulin sensitivity index and PKB Ser473 phosphorylation, with VO_2max_ or fat oxidation during exercise explaining 10–13% of the variance in insulin sensitivity index, independent of age, body composition and physical activity.

**Conclusions/Significance:**

These data indicate that reduced oxidative capacity and capacity for fatty acid utilisation at the whole body level are key features of the insulin resistant phenotype observed in South Asians, but that this is not the consequence of reduced skeletal muscle expression of oxidative and lipid metabolism genes.

## Introduction

South Asians have a high risk of diabetes, particularly when they migrate away from the Indian Subcontinent [1]–[4], with increased insulin resistance likely to play a key role [5]–[9]. For a given BMI, South Asians generally have higher percentages of body fat, increased waist-to-hip ratios and increased truncal skinfold thickness than European comparators [3], [10] – although interestingly increased visceral fat is not a consistent finding [7], [11] – and it has been suggested that this tendency to increased adiposity and central fat distribution contributes to the increased insulin resistance observed in this group. However, additional studies have shown that even after adjustment for BMI, waist-hip-ratio, and skin-fold thickness, insulin levels (both fasting and post glucose-load) remain significantly higher in South Asians [11], [12]. Furthermore, even when South Asian men are closely matched for BMI, waist-to-hip ratios and visceral fat areas with European men, they exhibit substantially increased insulin resistance [11]. In addition, South Asians develop diabetes and metabolic disturbances associated with insulin resistance at lower BMI values than Europeans [13]. Thus, it appears that the increased insulin resistance in South Asians cannot be fully explained by differences in adiposity and/or abdominal fat accumulation.

Accumulation of lipid within skeletal muscle – particularly of active lipid intermediates such as long chain fatty acyl-CoA, diacylglycerol and ceramide – is likely to play a causal role in insulin resistance [14]. It has been reported that South Asians have 30% higher intramuscular triglyceride (IMTG) concentrations than BMI-matched Europeans [11]. Although it is now generally accepted that IMTG *per se* (as opposed to lipid intermediates) is unlikely to play a direct role in insulin resistance, IMTG does provide a useful marker of cytosolic lipid accumulation [14], thus the observation of elevated IMTG in South Asians is suggestive of a deficiency in skeletal muscle lipid metabolism. Accumulating evidence indicates that defects in skeletal muscle oxidative capacity and low rates of skeletal muscle lipid oxidation are likely to contribute to skeletal muscle lipid accumulation and consequent insulin resistance [15]–[17]. However, Nair and colleagues reported that middle-aged non-diabetic Asian Indians had increased skeletal muscle expression of genes involved with oxidative phosphorylation and the citrate cycle and increased capacity for mitochondrial ATP production than matched Americans of European descent, despite being more insulin resistant, concluding that mitochondrial dysfunction could not account for the Asian Indians' greater insulin resistance [9]. However, these data may not tell the whole story. In contrast to the skeletal muscle data indicating increased mitochondrial capacity, the available evidence at the whole-body level indicates that South Asians have lower maximal oxygen uptake (VO_2max_) values – an index of oxidative capacity at the whole-body level – than matched European comparators [12], [18] and VO_2max_ is a strong independent predictor of whole body insulin sensitivity [19], [20]. However, while it is known that cardiorespiratory fitness is closely associated with skeletal muscle lipid oxidative capacity [21]–[23], it is not known whether capacity for lipid oxidation is reduced in South Asians compared to Europeans, or whether these factors contribute to South Asians' increased insulin resistance.

Thus, in order to gain further insight into the potential role of altered capacity for lipid oxidation in mediating the South Asian insulin resistant phenotype, we undertook a detailed investigation of oxidative capacity and capacity for fatty acid utilisation in matched South Asian and European men using a range of whole-body and skeletal muscle measures. In addition, we characterised the expression of insulin signalling molecules in skeletal muscle biopsies, to determine whether alterations in key signalling proteins were associated with alterations in oxidative capacity and/or insulin sensitivity.

## Methods

### Subjects and recruitment

Twenty men of South Asian ethnic origin and 20 men of white European decent, individually matched for age (±5 years) and BMI (±2 kg.m^−2^) were recruited via a study website and local advertising. Data from one European man was excluded as he was subsequently discovered to have diabetes: the final study group therefore contained 20 South Asian and 19 European men. Anthropometric, physiological and biochemical characteristics of the volunteers included in the analyses are shown in Tables 1, 2 and 3. All volunteers currently resided in Glasgow; 18 of the European and 4 of the South Asian men had lived in the UK all of their lives. Of the 16 South Asians born outside the UK, mean (± SD) duration of UK residence was 2.5±5.0 years. All participants reported low to moderate levels of physical activity (<2 hours of planned exercise per week and physically inactive job), and were non-smokers, aged 18–40 years, in generally good health, with blood pressure <160/90 mmHg, and no known history of diabetes or cardiovascular disease. Other than one European using steroid and β2 agonist inhalers for asthma and topical steroids for eczema and another European using topical steroids for eczema, no volunteer was taking any medications. The study was approved by the North Glasgow NHS Trust Research Ethics Committee and was conducted according to the principles expressed in the Declaration of Helsinki. All participants gave written informed consent.

**Table 1:** Anthropometric data for European and South Asian men.

	South Asians(n = 20)	Europeans(n = 19)	P (unadjusted)	P (age and BMI adjusted)	P (age, BMI and fat mass adjusted)
Age (years)	26.9±3.9	24.5±5.5	0.12	**-**	**-**
BMI (kg.m^−2^)	23.6±2.9	22.6±2.7	0.31	**-**	**-**
Body mass (kg)	71.8±10.1	72.5±8.8	0.82	0.13	**0.034**
Height (cm)	**174.4±7.1**	**179.0±6.8**	**0.046**	0.11	**0.028**
Total fat mass (kg)	**18.4±5.3**	**13.6±5.2**	**0.007**	**<0.0005**	**-**
Trunk fat mass (kg)	**10.0±3.3**	**7.1±3.3**	**0.009**	**<0.0005**	0.520
Arm fat mass (kg)	**1.4±0.5**	**1.0±0.5**	**0.02**	**0.008**	0.606
Leg fat mass (kg)	**6.3±1.6**	**5.0±1.5**	**0.009**	**0.004**	0.618
Fat-free mass (kg)	**53.4±6.6**	**58.9±5.9**	**0.010**	**0.001**	**0.034**
Total lean mass (kg)	**50.0±5.8**	**56.3±5.6**	**0.001**	**<0.0005**	**0.009**
Trunk lean mass (kg)	**22.7±2.8**	**26.4±2.6**	**<0.0005**	**<0.0005**	**0.003**
Arm lean mass (kg)	6.2±0.8	6.5±0.9	0.25	0.08	0.327
Leg lean mass (kg)	**17.5±2.3**	**19.5±2.1**	**0.007**	**0.002**	**0.026**
Waist circumference (cm)	82.3±7.5	78.8±6.8	0.14	0.30	0.778
Hip circumference (cm)	97.3±5.8	96.6±1.1	0.69	0.59	0.463

Values are mean ± SD.

**Table 2:** Fitness and physical activity data for European and South Asian men.

	South Asians(n = 20)	Europeans(n = 19)	P (unadjusted)	P (age and BMI adjusted)	P (age, BMI and fat mass adjusted)
VO_2max_(ml.kg^−1^.min^−1^)	**40.6±6.6**	**52.4±5.7**	**<0.0005**	**<0.0005**	**0.001**
VO_2max_(ml.kg^−1^ fat-free mass.min^−1^)	**54.1±6.6**	**64.3±5.8**	**<0.0005**	**<0.0005**	**0.001**
Physical activity*(total MET-mins.day^−1^)	254±114	290±229	0.75	0.46	0.59

Values are mean ± SD.

*statistical analysis performed on log transformed data**.**

**Table 3:** Metabolic data for European and South Asian men.

	South Asians(n = 20)	Europeans(n = 19)	P (unadjusted)	P (age and BMI adjusted)	P (age, BMI and fat mass adjusted)
Fasting glucose*(mmol.l^−1^)	5.14±0.47	5.24±0.52	0.53	0.50	0.94
Fasting insulin*(mU.l^−1^)	**6.56±3.53**	**5.39±4.20**	0.11	0.17	**0.023**
Fasting NEFA*(mmol.l^−1^)	0.41±0.13	0.44±0.57	0.85	0.99	0.85
2 hour insulin*(mU.l^−1^)	**46.6±29.6**	**27.5±5.3**	**0.017**	**0.031**	**0.043**
Insulin AUC*(mU.l^−1^.min)	**6325±720**	**4374±661**	**0.036**	**0.045**	0.099
Glucose AUC*(mU.l^−1^.min)	833±32	885±39	0.35	0.46	0.74
NEFA AUC*(mU.l^−1^.min)	30.8±2.1	31.1±3.9	0.38	0.39	0.60
Insulin sensitivity index†	**5.89±2.93**	**7.96±3.49**	**0.048**	**0.047**	**0.012**
Fasting total cholesterol (mmol.l^−1^)	4.46±0.89	4.07±0.85	0.17	0.23	0.37
Fasting HDL cholesterol (mmol.l^−1^)	**1.08±0.22**	**1.37±0.20**	**<0.0005**	**<0.0005**	**0.001**
Fasting TG*(mmol.l^−1^)	1.18±0.65	0.87±0.56	0.054	0.11	0.13
Resting metabolic rate(kJ.kg fat-free mass^−1^.day^−1^)	114.1±9.0	118.6±10.8	0.170	0.378	0.059
Resting fasted fat oxidation(mg.kg^−1^ fat-free mass.min^−1^)	1.47±0.44	1.38±0.43	0.548	0.510	0.862

Values are mean ± SD.

*statistical analysis performed on log transformed data,

† statistical analysis performed on square-root transformed data.

### Body composition assessment

Dual X-Ray Absorptiometry (DEXA) scans (LUNAR Prodigy DEXA scanner, GE Healthcare Diagnostic Imaging, Slough, UK) were used to determine body composition and fat distribution. Height, body mass, waist and hip circumferences were also determined using standard protocols [24].

### Exercise tests

Exercise tests, undertaken following a 12-hour overnight fast, were performed to determine VO_2max_ and rates of fat oxidation during sub-maximal exercise. Following an exercise tolerance test to ensure no cardiovascular contraindications to maximal exercise [25], VO_2max_ was assessed using the modified Taylor incremental treadmill test protocol [26], with 1-minute expired air samples taken continuously using Douglas Bags and heart rate monitored by short-range telemetry. Achievement of VO_2max_ was verified by volitional exhaustion together with a rate of perceived exertion (RPE) of 19–20 [27], a respiratory exchange ratio >1.15, and heart rate within 10 beats of age-predicted maximum.

On a separate day subjects underwent a submaximal incremental treadmill test at 5.5 km.h^−1^, with an initial gradient calculated to elicit an oxygen uptake of ∼40% VO_2max_. Gradient increased by 1% every 4 minutes, until an RPE of 15–16 was achieved. Expired air, heart rate and RPE were taken during the final minute of each stage. Fat and carbohydrate oxidation rates at each exercise stage was determined from VO_2_ and VCO_2_ measurements in expired air by indirect calorimetry [28]. As skeletal muscle capacity for mitochondrial fat oxidation correlates strongly with fat oxidation during sub-maximal exercise [21], this test was used to provide an index of skeletal muscle fat oxidative capacity.

### Determination of habitual physical activity and diet

Habitual physical activity was assessed using the long form of the International Physical Activity Questionnaire [29] and habitual diet was assessed using a 120-item food-frequency questionnaire [30].

### Metabolic assessment and muscle biopsy

For these measurements volunteers attended the laboratory after 12-hour overnight fast, and at least a 60-hour abstention from planned exercise. Following a 10-minute rest lying on a couch, a 20-minute expired air sample was collected using a ventilated hood system (Oxycon Pro, Jaeger, Germany). Metabolic rate and rates of fat and carbohydrate oxidation were calculated by indirect calorimetry [28], assuming urinary nitrogen excretion to be 0.11 mg.kg^−1^.min^−1^ [31], [32]. A fasting blood sample was then taken, followed by a muscle biopsy from the vastus lateralis of the right leg, 20 cm above the patella, under local anaesthesia using a ‘semi-open’ technique [33]. Visible fat and connective tissue was removed with sterile forceps and samples were divided in six pieces. Four pieces were immediately snap-frozen in liquid N_2_, the remaining two pieces were incubated for 15 minutes in Krebs Ringer Hepes buffer (118 mM NaCl, 25 mM Hepes-NaOH, pH 7.4, 5 mM NaHCO_3_, 4.7 mM KCl, 1.2 mM MgSO_4_, 1.2 mM NaH_2_PO_4_, 2.5 mM CaCl_2_, 5mM glucose, 0.1% (w/v) bovine serum albumin) at 37°C, gassed with O_2_ prior to incubation for 10 min in the presence or absence of 10 nM human soluble insulin (Actrapid, Novo Nordisk), before snap-freezing in liquid N_2_ prior to homogenisation for immunoblotting analysis. The 10 nM insulin dose was chosen to maximally stimulate insulin signalling [34]. Subjects then consumed 75 g glucose orally in a 300 ml volume, with further blood samples taken at 30-minute intervals for 120 minutes.

### Plasma analyses

Blood was collected into potassium EDTA tubes, placed on ice, separated within 15 minutes and stored at -80°C until analysis. Glucose, TG, non-esterified fatty acid (NEFA), total cholesterol, and HDL cholesterol concentrations were determined by enzymatic colorimetric methods using commercially available kits (Roche Diagnostics Gmbh, Mannheim, Germany; Wako Chemicals GmbH, Neuss, Germany and Randox Laboratories Ltd., Co. Antrim, Ireland). Insulin was determined using a commercially available ELISA with <0.01% cross-reactivity with pro-insulin (Mercodia AB, Uppsala, Sweden). Adiponectin, leptin, IL-6 and TNFα concentrations were also determined by ELISA (R&D Systems Europe, Abingdon, United Kingdom).

### Determination of skeletal muscle expression of oxidative and lipid metabolism genes

RNA was extracted from muscle using an E.Z.N.A. Tissue RNA kit (Omega Bio-Tek, Inc., Norcross, GA) according to the manufacturer's protocol using Precellys Ceramic Homogenisation beads (CK14, PEQLAB Ltd, Farnborough, UK) and a Hybaid Ribolyser (Thermo Scientific, Loughborough, United Kingdom). cDNA was synthesised using an Applied BioSystems High Capacity cDNA Reverse Transcription kit (Life Technologies Corporation, Carlsbad, CA) with random hexamers according to the manufacturers protocol, but doubling the volumes to increase yield. Quantitative polymerase chain reaction (qPCR) assays were designed for each transcript (Table S1) using the Roche Universal ProbeLibrary Assay Design Center (http://www.roches-applied-science.com/) and mRNA expression levels of transcripts were determined as described previously [35].

### Determination of skeletal muscle mitochondrial DNA (mtDNA) to nuclear DNA (nDNA) ratio

DNA was extracted from muscle using a QIAamp DNA Mini Kit (Qiagen Ltd., Crawley, United Kingdom) according to the manufacturer's protocol (including the RNase treatment) using Precellys Ceramic Homogenisation beads (CK14, PEQLAB Ltd, Farnborough, UK) and a Hybaid Ribolyser (Thermo Scientific, Loughborough, United Kingdom). qPCR assays were designed, using the Roche Universal ProbeLibrary Assay Design Center, for the mitochondrial genome overlapping two neighbouring genes (tRNA leucine 1 and NADH dehydrogenase 1) and the nuclear genome using the β2-adrenergic receptor gene promoter (Table S1). Relative DNA levels were determined in a similar fashion to the mRNA expression levels.

### Determination of skeletal muscle insulin signalling protein expression

Muscle biopsies were homogenised in 8 volumes of homogenisation buffer (50 mM Tris-HCl, pH 7.4 at 4°C, 250 mM sucrose, 1 mM EDTA, 1 mM EGTA, 5 mM NaF, 5 mM Na_4_P_2_O_7_, 1 mM dithiothreitol, 1 mM Na_3_VO_4_, 0.1 mM benzamidine, 0.1 mM phenylmethylsuplhonyl fluoride, 5 µg.ml^−1^ soybean trypsin inhibitor) by 20 passes in a Dounce homogeniser at 4°C. Homogenates were centrifuged (350,000 g, 30 min, 4°C) to obtain cytosolic supernatant fractions. Pellets were re-suspended in 150 µl homogenisation buffer supplemented with 1% (v/v) NP-40 and incubated on ice for 30 min prior to centrifugation (100,000 g, 30 min, 4°C) to obtain microsomal supernatant fractions. Cytosolic and microsomal protein concentrations were assessed according to the method of Bradford [36].

Equal amounts of cytosolic/microsomal fraction protein were resolved by SDS-PAGE and subjected to immunoblotting using rabbit anti-IRS-1 (New England Biolabs, Hitchin, Hertfordshire, UK), mouse anti-phosphatidylinositol 3′-kinase (PI3K) p85 subunit (BD Biosciences, Oxford, Oxfordshire, UK), rabbit anti-PI3K p110β subunit and anti-PKCβ1 (Santa Cruz Biotechnology, Santa Cruz, CA, USA), mouse anti-glyceraldehyde 3-phosphate dehydrogenase (GAPDH) (Applied Biosystems, Warrington, Cheshire, UK), rabbit anti-PKB and anti-phospho-PKB Ser473 (New England Biolabs) antibodies. Blots were scanned and band intensity analysed using Image J software, normalised by comparison with an internal control human muscle lysate.

### Data analysis

Insulin sensitivity was calculated from fasting and post-glucose plasma glucose and insulin concentrations using the Insulin Sensitivity Index (ISI) derived by Matsuda and DeFronzo – i.e. 10000/√[(fasting glucose x fasting insulin) x (mean glucose during OGTT x mean insulin during OGTT)], which correlates highly with whole-body glucose disposal rate during a euglyaemic hyperinsulinaemic clamp [37].

Data were analysed using Statistica (version 6.0, StatSoft Inc., Oklahoma) and Minitab (version 14, Minitab Inc., Pennsylvania). Data were tested for normality using the Ryan-Joiner normality test and transformed as appropriate (determined using Box-Cox plots). General linear models were used to compare data between European and South Asian groups. As age and BMI were not identical between the two groups (although not significantly different), statistical analysis was undertaken both unadjusted and adjusted for age and BMI. Further adjustment was undertaken as appropriate to determine independence from potential confounders. Regression analysis was used to determine relationships between variables. A homogeneity-of-slopes regression model was used to identify whether the slope of relationships between variables differed between the South Asian and European groups. Where the slopes differed significantly between the two groups, univariate regressions were performed for the South Asian and European groups separately, otherwise univariate regressions were performed on the combined group to maximize statistical power. Partial correlations were undertaken where appropriate to adjust for the effect of potential confounders (such as age, BMI, fat mass and physical activity level) on relationships between variables. Statistical significance was accepted at the p<0.05 level.

## Results

### Anthropometric characteristics

Anthropometric data are presented in Table 1. There were no significant differences in age, BMI or body mass between South Asians and Europeans, but the South Asians were shorter; had greater total, trunk, arm and leg fat mass; and lower fat-free mass, and total, trunk and leg lean mass than the Europeans. These differences persisted after adjustment for age and BMI. Further adjustment for fat mass abolished differences in trunk, arm and leg fat between groups, indicating no significant differences in regional distribution between Europeans and South Asians.

### Physical activity and dietary data

Neither habitual physical activity levels (Table 2), nor energy (South Asians: 8.8±2.0 MJ.day^−1^, Europeans: 9.4±2.0 MJ.day^−1^, p = 0.41), fat (71±18 g.day^−1^ *vs* 80±26 g.day^−1^, p = 0.19), carbohydrate (290±90 g.day^−1^ *vs* 260±50 g.day^−1^, p = 0.20) or protein (87±17 g.day^−1^ *vs* 98±25 g.day^−1^, p = 0.12) intake differed significantly between the South Asian and European groups. However, self-reported alcohol intake was significantly higher in the Europeans (South Asians: 3±7 g.day^−1^, Europeans: 20±17 g.day^−1^, p = 0.0002).

### Exercise test data

**Figure 1:**
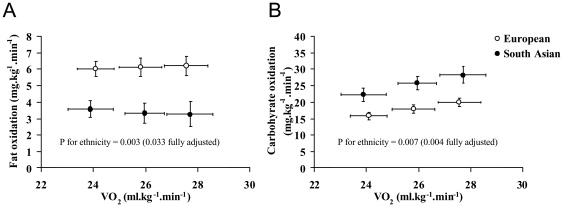
Fat oxidation (A) and carbohydrate oxidation (B) during incremental submaximal exercise. Intensity expressed in terms of absolute oxygen uptake (i.e. ml.kg^−1^.min^−1^). P values shown are for the main-effect difference between European and South Asian groups, either unadjusted, or adjusted for age, BMI and fat mass.

**Figure 2:**
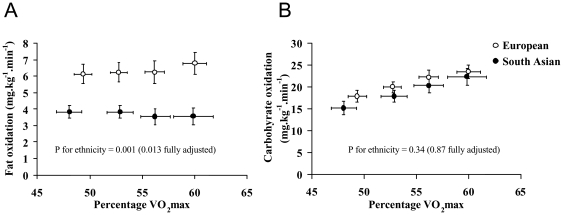
Fat oxidation (A) and carbohydrate oxidation (B) during incremental submaximal exercise. Intensity expressed relative to each individual's maximal oxygen uptake (i.e. percentage VO_2max_). P values shown are for the main-effect difference between European and South Asian groups, either unadjusted, or adjusted for age, BMI and fat mass.

Fitness and physical activity data are presented in Table 2 and Figures 1 and 2. South Asians had significantly lower VO_2max_ values than Europeans, irrespective of the units of expression. These differences remained significant after adjustment for age, BMI and fat mass. South Asians had lower rates of fat oxidation than Europeans during sub-maximal exercise, whether intensity was expressed in absolute terms (i.e. ml.kg^−1^.min^−1^) (Figure 1A) or relative to VO_2max_ (Figure 2A). This difference persisted after adjustment for age, BMI and fat mass. Carbohydrate oxidation rates in South Asians during sub-maximal exercise were similar to Europeans at the same relative exercise intensity (Figure 2B), and higher at the same absolute exercise intensity (Figure 1B).

### Metabolic data

**Figure 3:**
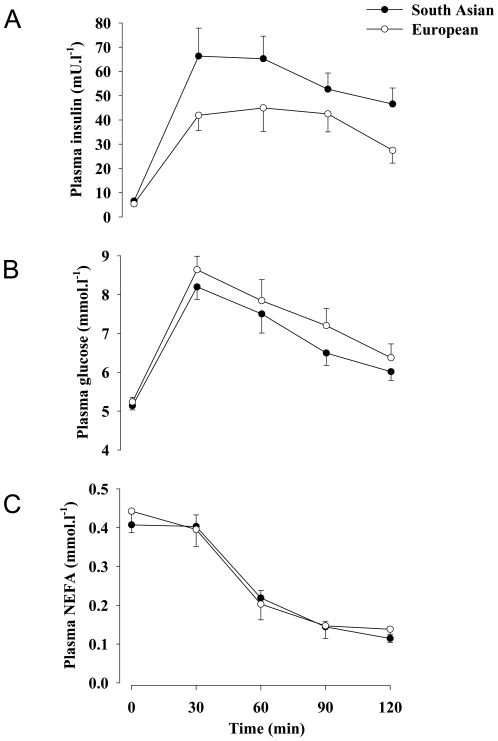
Plasma insulin (A), glucose (B) and NEFA (C) responses to a 75 g oral glucose load. Summary measures of these responses are presented in Table 3.

**Figure 4:**
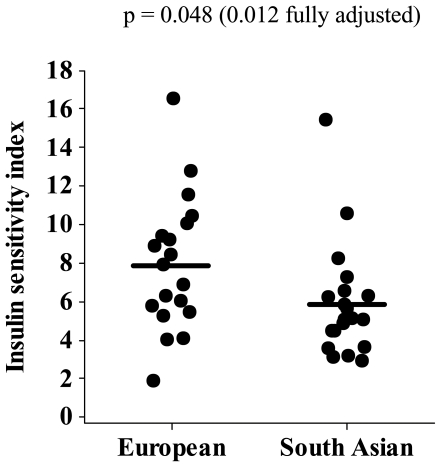
Individual insulin sensitivity index values for the European and South Asian men. Horizontal bars denote mean values. P values shown are for the difference between European and South Asian groups, either unadjusted, or adjusted for age, BMI and fat mass.

Metabolic data are presented in Table 3 and Figures 3 and 4. ISI and HDL-cholesterol were lower in South Asians than Europeans in unadjusted analysis; this persisted after adjustment for age, BMI and fat mass. Insulin AUC and 2-hour insulin were higher in South Asians than Europeans in unadjusted analysis and after adjustment for age and BMI. 2-hour insulin remained higher after further adjustment for fat mass. Although not different between groups in the unadjusted analysis, fasting insulin was higher in South Asians than Europeans after adjustment for age, BMI and fat mass. Adjustment for trunk fat mass, instead of total fat mass, did not alter the findings (data not shown). The significant difference in ISI (p = 0.044) and HDL-cholesterol concentrations (p = 0.004) between South Asian and Europeans was retained when data were adjusted for percentage body fat, rather than total fat mass, but with this adjustment the difference in fasting (p = 0.12) and 2-hour (p = 0.13) insulin concentrations between groups was lost. When ISI values were further adjusted for VO_2max_, or for the rate of fat oxidation fat oxidation during sub-maximal exercise at 55% VO_2max_, the significant difference between South Asian and European groups was abolished (p = 0.16 and 0.12, respectively). Resting metabolic rate and rate of fat oxidation (expressed per kg fat-free mass) did not differ between groups.

### Skeletal muscle mtDNA to nDNA ratio

The mtDNA to nDNA ratio in skeletal muscle did not differ between South Asians and Europeans (mean (95% CI) South Asians: 0.94 (0.71 to 1.21), Europeans: 1.13 (0.82 to 1.49), p = 0.39).

### Skeletal muscle expression of oxidative and lipid metabolism genes

Expression of oxidative and lipid metabolism genes in skeletal muscle is shown in Table 4. Expression of carnitine palmitoyltransferase 1A (CPT1A) was 1.79-fold greater (p = 0.023) and expression of fatty acid synthase (FASN) was 1.96-fold greater (p = 0.036) in South Asians than Europeans.

**Table 4:** Oxidative and lipid metabolism gene expression in skeletal muscle in European and South Asian men.

	South Asians(n = 20)	Europeans(n = 19)	P (unadjusted)	P (age and BMI adjusted)	P (age, BMI and fat mass adjusted)
CD36	1.15(0.94 to 1.41)	1.00(0.81 to 1.23)	0.343	0.210	0.168
CPT1A	**1.79** **(1.32 to 2.43)**	**1.00** **(0.69 to 1.46)**	**0.023**	**0.013**	**0.007**
CPT1B	1.22(0.89 to 1.68)	1.00(0.71 to 1.40)	0.406	0.244	0.088
CPT2	1.16(1.00 to 1.34)	1.00(0.74 to 1.36)	0.388	0.282	0.459
HADHA	1.25(0.98 to 1.59)	1.00(0.77 to 1.30)	0.233	0.078	**0.049**
HADHB	1.23(0.96 to 1.57)	1.00(0.75 to 1.33)	0.296	0.356	0.538
ACACA	1.32(0.86 to 2.04)	1.00(0.7 to 1.43)	0.342	0.201	0.109
ACACB	1.25(0.84 to 1.86)	1.00(0.69 to 1.46)	0.422	0.183	0.141
CS	1.10(0.84 to 1.44)	1.00(0.79 to 1.27)	0.606	0.303	0.066
CS (long transcript)	1.29(1.04 to 1.6)	1.00(0.8 to 1.26)	0.122	0.061	**0.031**
CS (short transcript)	1.09(0.79 to 1.5)	1.00(0.73 to 1.37)	0.712	0.323	0.064
COX1	1.07(0.72 to 1.59)	1.00(0.69 to 1.44)	0.805	0.515	0.447
FASN	**1.96** **(1.19 to 3.25)**	**1.00** **(0.72 to 1.39)**	**0.036**	**0.025**	0.111
FADS3	1.65(1.19 to 2.28)	1.00(0.64 to 1.56)	0.082	**0.037**	**0.023**

Values are mean (95% CI), expressed relative to mean value in European group.

### Skeletal muscle insulin signalling protein expression

**Figure 5:**
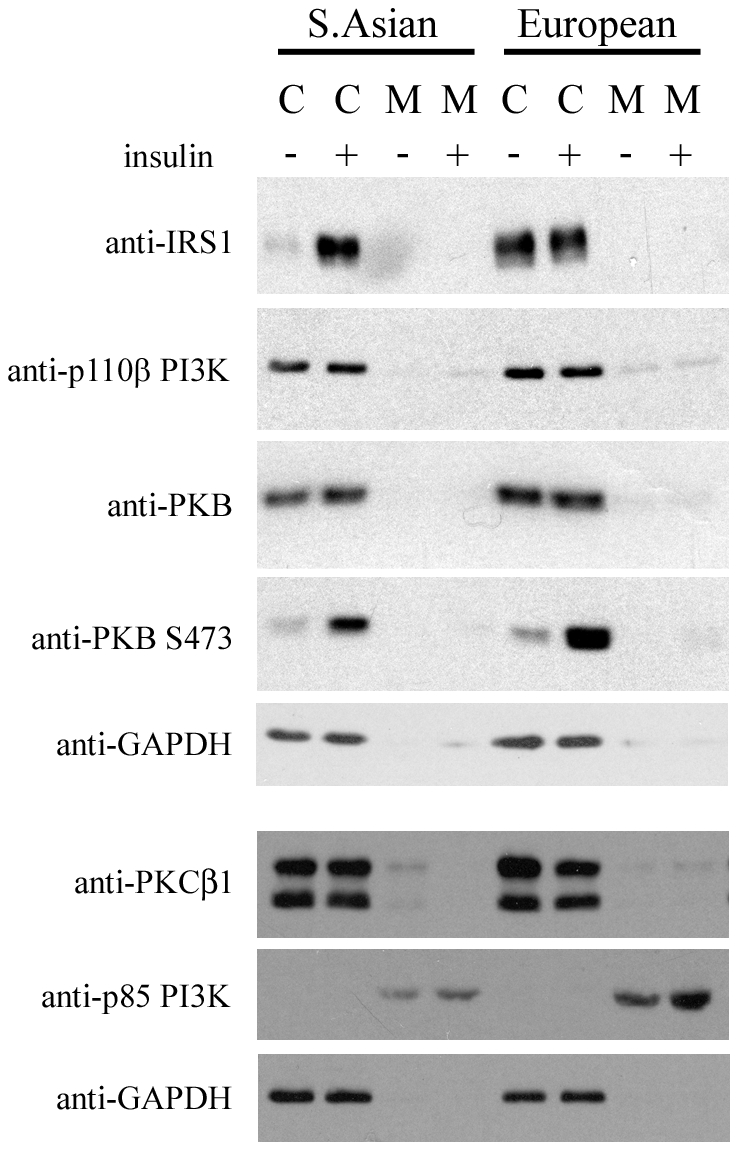
Insulin signalling molecule expression in subjects. Soluble cytosolic (C) and microsomal (M) fractions were prepared from muscles incubated *ex vivo* in the presence or absence of insulin. Fractions were resolved by SDS-PAGE and subjected to immunoblotting with the antibodies indicated. Representative blots are shown from one South Asian and one European subject.

Insulin signalling protein expression data (and n for each variable) are shown in Table 5. Representative blots are shown in Figure 5. Technical difficulties limited these data to a sub-set of participants, and one outlier (>9 SDs from mean) for p110β PI3K subunit expression was excluded as its inclusion prevented data normalization. Expression of IRS-1, PI3K (p110β), PKB and PKCβ1 was principally observed in the soluble cytosolic fraction, whereas expression of PI3K (p85) was principally observed in the microsomal fraction (Figure 5). Cytosolic IRS-1 protein expression was significantly lower in South Asians than Europeans in unadjusted analysis, but this difference was abolished on adjustment (Table 5). Microsomal expression of the PI3K p85 subunit was ∼50% lower in South Asians than Europeans; this remained significant after adjustment for age, BMI and fat mass (Table 5). Cytosolic PKB protein expression was also ∼50% lower in South Asians than Europeans. This difference remained after adjustment for age and BMI, and became borderline significant after further adjustment for fat mass (Table 5, p = 0.054). Neither protein expression of the PI3K p110β subunit, nor protein expression of cytosolic or microsomal PKCβ1 differed significantly between groups. *Ex vivo* stimulation of muscle with insulin had no effect on the expression of IRS-1, PI3K (p85 or p110β), PKB or PKCβ1 in microsomal or soluble cytosolic fractions (Figure 5). Basal PKB phosphorylation at Ser473 was over 60% lower in South Asians than Europeans; this remained significant after adjustment (Table 5). Insulin-stimulated PKB phosphorylation did not differ significantly between groups (Table 5). The ratio of PKB phosphorylation at Ser473 to PKB expression did not differ significantly between South Asians and Europeans under either basal or insulin-stimulated conditions.

**Table 5:** Skeletal muscle insulin signalling protein expression in South Asian and European men.

	South Asians	Europeans	P (unadjusted)	P (age and BMI adjusted)	P (age, BMI and fat mass adjusted)
IRS-1(n = 8 South Asians, 7 Europeans)	**0.35** **(0.13 to 0.56)**	**1.00** **(0.50 to 1.50)**	**0.028**	0.699	0.699
PI3K, p85 subunit(n = 8 South Asians, 8 Europeans)	**0.51** **(0.28 to 0.75)**	**1.00** **(0.84 to 1.16)**	**0.005**	0.079	**0.049**
PI3K, p110β subunit(n = 5 South Asians, 14 Europeans)	1.05(0.82 to 1.28)	1.00(0.78 to 1.22)	0.800	0.281	0.159
PKB*(n = 16 South Asians, 12 Europeans)	**0.51** **(0.36 to 0.71)**	**1.00** **(0.57 to 1.75)**	**0.041**	**0.003**	0.054
Basal PKB Ser473 phosphoryation*(n = 14 South Asians, 11 Europeans)	**0.38** **(0.27 to 0.52)**	**1.00** **(0.74 to 1.38)**	**<0.0005**	**<0.0005**	**0.019**
Insulin-stimulated PKB Ser473 phosphoryation*(n = 14 South Asians, 11 Europeans)	1.58(1.22 to 2.05)	1.63(1.07 to 2.48)	0.889	0.419	0.287
Cytosolic PKCβ1(n = 8 South Asians, 9 Europeans)	0.94(0.61 to 1.27)	1.00(0.64 to 1.37)	0.813	0.739	0.759
Microsomal PKCβ1(n = 8 South Asians, 9 Europeans)†	0.17(0.11 to 0.24)	0.20(0.05 to 0.46)	0.824	0.907	0.734

Values are mean (95% CI), expressed relative to mean value in European group.

*statistical analysis performed on log transformed data,

† statistical analysis performed on square-root transformed data.

### Anthropometric and physiological correlates with insulin sensitivity index and insulin signalling protein expression

The regression slopes for these relationships did not differ significantly between South Asians and Europeans so data are presented for the combined group. As expected, square-root ISI correlated significantly with BMI (r = −0.373, p = 0.019), waist circumference (r = −0.387, p = 0.015) and trunk fat mass (r = −0.359, p = 0.025). Square-root ISI correlated significantly with height (r = 0.329, p = 0.041). None of the other measured body composition variables significantly correlated with square-root ISI.

**Figure 6:**
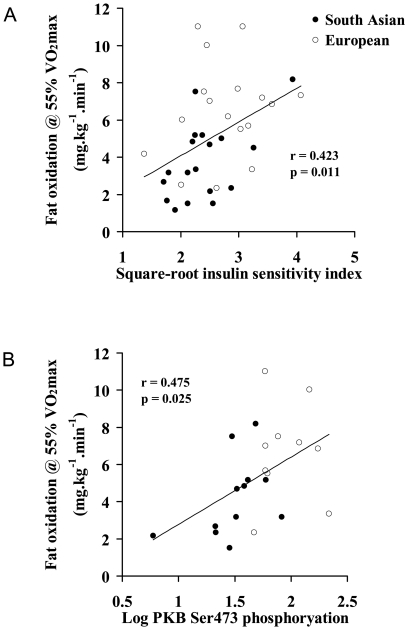
Relationship between exercise fat oxidation and insulin sensitivity index (A) and basal PKB Ser473 phosphoryation (B).

In unadjusted univariate correlations, VO_2max_, expressed in ml.kg^−1^.min^−1^ (r = 0.399, p = 0.012) or ml.kg^−1^ fat-free mass.min^−1^ (r = 0.352, p = 0.028) correlated significantly with square-root ISI. In addition, fat oxidation during sub-maximal exercise at 55% VO_2max_, expressed in mg.kg^−1^.min^−1^ (r = 0.423, p = 0.011) (Figure 6) or in mg.kg^−1^ fat-free mass.min^−1^ (r = 0.409, p = 0.015), and fat oxidation at an absolute VO_2_ of 25 mg.kg^−1^.min^−1^ (r = 0.370, p = 0.029) correlated significantly with square-root ISI. Adjustment for age, BMI, fat mass and physical activity slightly attenuated the relationships. However, even after adjustment significant relationships were observed between square root ISI, and VO_2max_ expressed in ml.kg^−1^ fat-free mass.min^−1^ (r = 0.318, p = 0.049) and fat oxidation during submaximal exercise at 55% VO_2max_, expressed in mg.kg^−1^.min^−1^ (r = 0.337, p = 0.048) or in mg.kg^−1^ fat-free mass.min^−1^ (r = 0.358, p = 0.035). Thus, independent of age, body composition and physical activity level, VO_2max_ or fat oxidation during submaximal exercise explained 10-13% of the variance (i.e. r^2^) in square-root ISI.

Neither fat oxidation rate at rest nor resting metabolic rate (however expressed) correlated significantly with square-root ISI. In addition, fat oxidation rate at rest did not correlate significantly with fat oxidation rates during exercise (however expressed).

**Figure 7:**
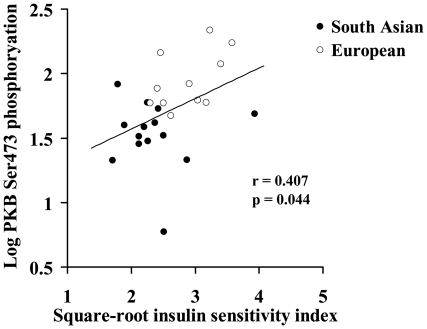
Relationship between basal PKB Ser473 phosphoryation and insulin sensitivity index.

Basal PKB phosphorylation at Ser473 correlated significantly with square-root ISI (r = 0.407, p = 0.044) (Figure 7), and indices of body composition (significant negative correlations with total, arm, leg and trunk fat mass, and waist (r = −0.401 to −0.440, all p<0.05); and significant positive correlations with fat-free mass and total, leg and trunk lean mass (r = 0.538 to 0.546, all p<0.05)). In addition, basal PKB Ser473 phosphorylation was strongly correlated with VO_2max_, expressed in ml.kg^−1^.min^−1^ (r = 0.634, p = 0.001) or ml.kg^−1^ fat-free mass.min^−1^ (r = 0.539, p = 0.005); fat oxidation during exercise at 55% VO_2max_, expressed in mg.kg^−1^.min^−1^ (r = 0.475, p = 0.025) (Figure 4); and fat oxidation at an absolute VO_2_ of 25 mg.kg^−1^.min^−1^ (r = 0.588, p = 0.004). There was a borderline significant correlation between basal Ser473 PKB phosphorylation and fat oxidation during exercise at 55% VO_2max_, expressed in mg.kg^−1^ fat-free mass.min^−1^ (r = 0.406, p = 0.061). Although there was no relationship between basal and insulin-stimulated Ser473 PKB phosphorylation (r = 0.080, p = 0.705), and insulin-stimulated Ser473 PKB phosphorylation did not differ significantly between the South Asian and European groups, insulin-stimulated PKB phosphorylation at Ser473 also correlated significantly with square-root ISI (r = 0.427, p = 0.033) and indices of body composition (negative correlations with total and trunk fat mass, BMI and waist (r = −0.456 to – 0.533, all p<0.05)). However, insulin-stimulated Ser473 PKB phosphorylation did not correlate significantly with VO_2max_ or fat oxidation during exercise, however expressed. None of the other measured insulin signalling proteins correlated significantly with square-root ISI, or any indices of body composition, but protein expression of IRS-1 correlated significantly with VO_2max_, expressed in ml.kg^−1^.min^−1^ (r = 0.677, p = 0.006) or ml.kg^−1^ fat-free mass.min^−1^ (r = 0.649, p = 0.009).

### Relationships between skeletal muscle and whole-body metabolism

Homogeneity-of-slopes regression analysis revealed significant interactions between South Asians and Europeans in the slope of the relationships between expression of a number of skeletal muscle genes and whole-body indices. South Asian and European groups were therefore considered separately in these analyses. There was a significant negative correlation between square-root ISI and log CPT1A gene expression in South Asians (r = − 0.533, p = 0.016), but not in Europeans (r = 0.035, p = 0.886). Expression of none of the other measured oxidative and lipid metabolism genes correlated significantly with square-root ISI in either group.

**Figure 8:**
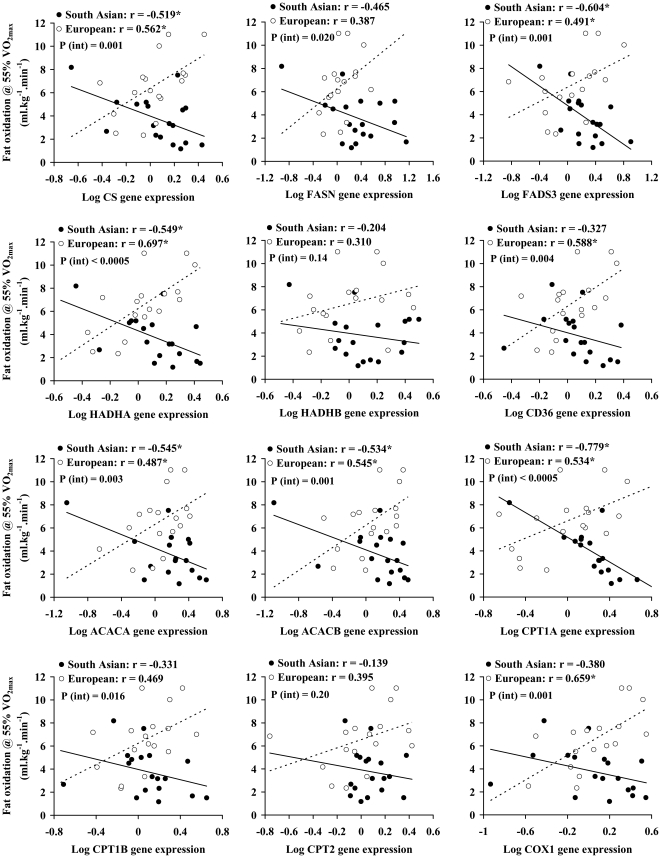
Relationships between exercise fat oxidation and skeletal muscle expression of oxidative and lipid metabolism genes. Significant correlations (p<0.05) are denoted by an asterix. P (int) signifies p-value for interaction in relationship between gene expression and fat oxidation during exercise between European and South Asian groups.

Figure 8 shows the relationships between fat oxidation during exercise at 55% VO_2max_, expressed in mg.kg^−1^.min^−1^ and skeletal muscle expression of oxidative and lipid metabolism genes in the South Asian and European groups. In general, positive associations were seen between skeletal muscle gene expression and fat oxidation during exercise in the Europeans, with significant positive correlations observed with fat oxidation during exercise and skeletal muscle CS, FASD3, HADHA, CD36, ACACA, ACACB, CPT1A and COX1 gene expression (r = 0.487 to 0.697, all p<0.05). In contrast negative association were seen in South Asians between fat oxidation during exercise and skeletal muscle CS, FADS3, HADHA, ACACA, ACACB and CPT1A (r = −0.519 to −0.779, all p<0.05). For 10 of the 12 genes investigated, a significant interaction was observed between the European and South Asian groups in the relationship between skeletal muscle gene expression and fat oxidation during exercise (Figure 8). Expressing fat oxidation during exercise in mg.kg^−1^ fat-free mass.min^−1^ yielded essentially the same results.

There were no significant correlations between skeletal muscle expression of any oxidative and lipid metabolism gene and VO_2max_ in either the South Asian or the European group.

## Discussion

We report four main novel findings from this study: 1) that South Asians oxidised less fat during submaximal exercise than Europeans, a difference which persisted after adjustment for age, BMI and fat mass; 2) that South Asians had reduced skeletal muscle expression of key insulin signalling proteins than Europeans; 3) that VO_2max_ and fat oxidation during submaximal exercise correlated significantly with whole-body insulin sensitivity (and PKB Ser473 phosphorylation), independently of age and body composition; and 4) that the relationship between fat oxidation during submaximal exercise and skeletal muscle expression of oxidative and lipid metabolism genes differed between South Asians and Europeans. Collectively, these data indicate that reduced oxidative capacity and capacity for fatty acid utilisation at the whole-body level are key features of the insulin resistant phenotype observed in South Asians, but that this is not the consequence of reduced skeletal muscle expression of oxidative and lipid metabolism genes.

Low VO_2max_ values are often [19], [20], [38], but not always [38], associated with insulin resistance in white European and American populations, and low cardiorespiratory fitness is an independent predictor of type 2 diabetes risk [39]. Cardiorespiratory fitness is closely associated with skeletal muscle lipid oxidative capacity [21]−[23] and it is likely that fitness influences insulin sensitivity, at least in part, via effects on muscle lipid metabolism [40], [41]. In the present report, adjusting ISI values for either VO_2max_ or rate of fat oxidation during exercise abolished the difference in insulin sensitivity between the South Asian and European groups, and strong correlations were evident between VO_2max_ and fat oxidation during exercise (r = 0.58 to 0.67, depending on units of measurement, all p<0.0005). This supports the suggestion that low oxidative capacity/capacity for fatty acid utilisation is a central feature of the South Asian insulin resistance phenotype, and highlights the fact that the lower cardiorespiratory fitness and reduced capacity to oxidize fat in this group are likely to largely reflect the same underlying mechanism. Interestingly, while South Asians oxidised ∼40% less fat than Europeans during submaximal exercise, fat oxidation rates did not differ between the groups at rest, and fat oxidation during exercise, but not at rest, correlated with body insulin sensitivity. There are two potential reasons for this. Firstly, whole-body fat oxidation during exercise largely reflects fat oxidation in skeletal muscle. In the present study, fat oxidation increased ∼3−6 fold during exercise compared to rest and this increase would almost exclusively be attributable to changes in skeletal muscle. In contrast, skeletal muscle only contributes about 20−30% of resting energy expenditure [42], thus whole-body fat oxidation at rest is to a large degree determined by tissues other than skeletal muscle. Thus, if skeletal muscle, rather than whole-body, fat oxidation is the key regulator of insulin sensitivity, the relationship would be more clearly revealed in whole-body measurements made during exercise rather than at rest. Secondly, it is possible that a deficiency in the capacity of muscle to oxidise fat becomes more evident when energy demand is high, and is thus revealed during exercise. These findings highlight the value of exercise testing in providing insights into muscle metabolism in a relatively non-invasive manner.

However, in contrast to the observations made at the whole-body level, South Asians did not exhibit lower expression of oxidative and lipid metabolism genes in skeletal muscle biospies than Europeans, and indeed expression of CPT1A and FASN were higher in South Asians. In addition, other than a negative correlation between expression of CPT1A and ISI in the South Asians – the opposite direction of the expected association – expression of none of these genes was related to whole-body insulin sensitivity. Furthermore, the mtDNA to nDNA ratio, which provides an index of mitochondrial biogenesis [43], did not differ between the two groups. Our data therefore indicate that reduced skeletal muscle expression of oxidative and lipid metabolism genes does not explain the increased insulin resistance observed in South Asians. These findings are broadly consistent with those of Nair and colleagues reported that Asian Indians living in the USA had increased skeletal muscle capacity for oxidative phosphorylation and a higher mitochondrial DNA copy number than age- and BMI-matched men of Northern European descent, concluding that mitochondrial dysfunction could not account for the Asian Indians' greater insulin resistance [9]. Thus, it seems likely that the reduced capacity of South Asians to oxidise fat during exercise represents a defect in substrate delivery to muscle, rather than in mitochondrial capacity to oxidise lipids. In Europeans, capacity for mitochondrial fat oxidation in *ex vivo* muscle biopsy samples has been shown to correlate strongly with whole-body fat oxidation during sub-maximal exercise (but not at rest) [21]; this is consistent with observations in the present study where positive correlations were observed between skeletal muscle expression of oxidative and lipid metabolism genes and fat oxidation during submaximal exercise in the European group. However, strikingly, this pattern was reversed in the South Asian group where negative correlations were observed between skeletal muscle gene expression and fat oxidation during exercise, and significant interactions in the relationships between skeletal muscle gene expression and fat oxidation during exercise were evident between the European and South Asian groups. Thus, the South Asians with the highest expression of oxidative and lipid metabolism genes oxidised the least fat during exercise. Conceivably, this might reflect a compensatory adaptation within muscle in response to impaired microvascular perfusion leading to reduced fuel delivery. While we have no data from the present study to support this suggestion, South Asian men have been shown to have impaired endothelial function in forearm resistance vessels [44] and reduced nitric oxide bioavailability both at rest and during exercise [45], which would be consistent with this hypothesis. Furthermore, there is clear evidence that skeletal muscle oxidative capacity [46]−[48], capacity for fat utilisation [47], [49] and insulin sensitivity [50]−[52], are governed not only by mitochondrial function, but also capillary density and recruitment. Clearly, further study is now needed to determine whether the increased insulin resistance and reduced ability to oxidise fat in South Asians, is a consequence of impairments in vascular function.

It is important to recognise that it is the mismatch between fatty acid mobilisation and oxidation, rather than a low ability of muscle to oxidise fat *per se*, which can lead to insulin resistance. Recent studies have shown improvements in insulin sensitivity with weight loss (which reduces fatty acid mobilisation [53]), in the absence of changes in oxidative capacity or fatty acid oxidation rates [53]−[55], and have shown that increasing fatty acid mobilisation by lipid infusion can abolish improvements in insulin sensitivity elicited by exercise training, despite fatty acid oxidation rates remaining elevated [53]. Thus, capacity for fatty acid oxidation clearly only provides one part of the story, and further study is needed to determine whether differences in fatty acid mobilisation exist between South Asians and Europeans that may contribute to the greater insulin resistance in the former group.

To the best of our knowledge, there have been no previously published data comparing insulin signalling molecule expression between any ethnic groups. Our novel data reveal that, in unadjusted analysis, South Asians had significantly lower protein expression of IRS-1, the p85 subunit of PI3K, PKB and basal PKB Ser473 phosphorylation. IRS-1 is the principal IRS involved in muscle insulin-stimulated glucose transport [56]. However, it is unclear from the literature the extent to which altered IRS-1 expression is a characteristic of insulin resistant conditions in humans. While reduced IRS-1 expression has been reported in muscle of individuals with type 2 diabetes or obese subjects [57], [58], other studies have reported no difference in muscle IRS-1 expression in subjects with polycystic ovary syndrome (PCOS) [59] insulin resistance [60], or type 2 diabetes [61], implying that changes in the insulin signalling pathway downstream of IRS-1 may play a more important role in human insulin resistance. The present data imply reduced IRS-1 signalling in South Asians, which may manifest as impaired insulin-stimulated glucose transport and a degree of insulin resistance in the muscle, although this did not correlate significantly with our whole-body measurement of insulin sensitivity. However, it is probable that this reduced IRS-1 expression is simply a consequence of the greater adiposity in the South Asian group, rather than a fundamental difference in the properties of South Asians' skeletal muscle, as the difference was abolished after adjustment for adiposity.

Our data also revealed lower expression of p85, yet unaltered expression of p110β subunit of PI3K in the muscles of South Asians compared to Europeans. Furthermore, the difference persisted after adjustment for age, BMI and fat mass, implying a fundamental difference in these aspects of insulin signalling between South Asian and European skeletal muscle. The implications, however, of altered PI3K p85 subunit expression on insulin resistance are somewhat unclear in the literature, in which the majority of studies have reported *increased* p85 expression to be associated with insulin resistance in human muscle and animals [62]−[65] and downregulation of p85 has been reported to reverse the inhibition of insulin-stimulated glucose transport in 3T3-L1 adipocytes caused by constitutively active PKB [66]. In contrast, reduced p85 expression has also been reported to be associated with obesity in a single study in placenta [67]. The reduction observed in South Asians in the current study is, therefore, in disagreement with many studies of insulin-resistant cohorts, yet this may reflect the requirement for an optimal level of p85 expression, whereby increased or decreased expression both have detrimental consequences in terms of insulin sensitivity.

PKB exists as three isoforms, α (Akt1), β (Akt2) and γ (Akt3), and studies in knockout mice have indicated that PKBβ is the principal isoform regulating glucose homeostasis [68], [69]. Impaired insulin-stimulated PKB phosphorylation/activity has been observed in skeletal muscle from subjects with type 2 diabetes or PCOS [70]−[72], yet other studies have reported no significant difference in insulin-stimulated PKB activity or phosphorylation in skeletal muscle of subjects with type 2 diabetes [63], [73], [74]. In the present study, PKB expression and phosphorylation at Ser473 was assessed with antibodies that do not distinguish between the different isoforms. Intriguingly, PKB protein expression was ∼50% lower in South Asians than Europeans, although adjustment for age, BMI and fat mass, reduced this to borderline statistical significance (p = 0.054). Furthermore, basal Ser473 phosphorylation of PKB was over 60% lower in South Asians, a difference that persisted after adjustment for age, BMI and fat mass. In addition, expression correlated significantly with whole-body insulin sensitivity. The increased basal Ser473 phosphorylation observed in Europeans is unlikely to be a consequence of endogenous insulin associated with the muscle biopsies after washing, as South Asians exhibited higher fasting insulin concentrations. In addition, basal PKB serine 473 phosphorylation correlated strongly and significantly with whole-body fat oxidation rates during exercise, suggesting that differences in capacity for fat oxidation between Europeans and South Asians may influence insulin sensitivity via effects at this point in the insulin signalling pathway. In contrast to our findings on PKB, there were no differences between South Asian and European muscle in protein expression of cytosolic or microsomal PKC β1, which negatively regulates insulin signalling [75], suggesting that PKC β1 does not underlie the increased skeletal muscle insulin resistance observed in South Asians.

This study is not without limitations. We assessed insulin sensitivity from glucose and insulin responses to an oral glucose tolerance test, rather than using the gold-standard euglycaemic hyperinsulinaemic clamp. However, the insulin sensitivity index we used correlates well with clamp-derived measures of insulin sensitivity [37], has been widely accepted in the literature (>1000 citations in last decade), and was in our data set associated with skeletal muscle insulin signalling. In addition, we only assessed expression of insulin signalling proteins in the basal state – i.e. signalling protein expression in response to fasting insulin concentrations – and in response to maximal insulin stimulation. While the direction of the differences in expression in the basal data between South Asians and Europeans precludes potential confounding by differences in fasting insulin concentrations (South Asians had reduced signalling despite a tendency for higher insulin concentrations), this study does not provide data on insulin signalling across the range of physiological insulin doses and further study is needed to ascertain whether insulin signalling differs between South Asians and Europeans at insulin doses between basal and maximal. We also only measured gene expression of oxidative and lipid metabolism genes in skeletal muscle. None of these was lower in the South Asians than the Europeans, indicating that transcription of these genes is not defective in South Asian muscle, but further study is needed to determine whether differences in protein levels or activity of these enzymes exist between South Asian and European skeletal muscle. Finally, although the men were extensively phenotyped, we did not directly determine IMTG or skeletal muscle concentrations of lipid intermediates in this study and further investigation is needed to ascertain the extent to which these differ between South Asians and Europeans, and how they relate to capacity for fat oxidation during exercise and insulin sensitivity.

In summary, the present novel observations indicate that South Asians have reduced cardiorespiratory fitness and capacity for fat oxidation during exercise compared to matched Europeans, and these factors are associated with their lower insulin sensitivity, independent of adiposity, both at a whole body level and at the level of skeletal muscle PKB Ser473 phosphorylation. In particular, reduced basal PKB Ser473 phosphorylation appears to be an innate feature of South Asian, compared to European, muscle which is related to insulin resistance at the whole body level. However the reduced capacity for fat oxidation during exercise was not reflected in reduced skeletal muscle expression of oxidative and lipid metabolism genes, and the relationship between expression of these genes and whole-fat oxidation during exercise in South Asians was negative. This indicate that reduced skeletal muscle expression of oxidative and lipid metabolism genes does not explain the increased insulin resistance observed in South Asians, and further investigation is needed to elucidate the mechanisms underpinning the reduced capacity for fat oxidation in South Asians and how this relates to their reduced insulin sensitivity.

## Supporting Information

Table S1 Primer sequences and Universal ProbeLibrary Set probe numbers used for qPCR. Where more than one transcript is shown for an individual gene, the primers are common to all transcripts.
(0.10 MB DOC)
