# Supplementary material for: Fat Oxidation, Fitness and Skeletal Muscle Expression of Oxidative/Lipid Metabolism Genes in South Asians: Implications for Insulin Resistance?
Source: PLoS One. 2010 Dec 1;5(12):e14197. doi: 10.1371/journal.pone.0014197 (PMC2995737; doi:10.1371/journal.pone.0014197)
Supplement: Table S1 — Primer sequences and Universal ProbeLibrary Set probe numbers used for qPCR. Where more than one transcript is shown for an individual gene, the primers are common to all transcripts. (0.10 MB DOC) [file pone.0014197.s001.doc]

**Table S1.** Primer sequences and Universal ProbeLibrary Set probe numbers used for qPCR. Where more than one transcript is shown for an individual gene, the primers are common to all transcripts.

| **Gene Name and Ensembl ID** | **Ensembl Transcript IDs** | **Forward Primer**  ***Sequence 5’ to 3’*** | **Reverse Primer**  ***Sequence 5’ to 3’*** | **Probe No.** |
| --- | --- | --- | --- | --- |
| **Calibrator Genes** |  |  |  |  |
| **β-actin (ACTB)**  ENSG00000075624 | ENST00000331789 | ACTBSM0341F  ccaaccgcgagaagatga | ACTBSM0437R  ccagaggcgtacagggatag | 64 |
| **Glyceraldehyde-3-phosphate dehydrogenase**  **(GAPDH)**  ENSG00000111640 | ENST00000229239 | GAPDHCM0076F  ctctgctcctcctgttcgac | GAPDHCM0187R  acgaccaaatccgttgactc | 60 |
| **Low density lipoprotein receptor-related protein 10**  **(LRP10)**  ENSG00000197324 | ENST00000359591 | LRP10CM0747F  aggaacagactgtcaccatcag | LRP10CM0852R  gcctcacacagggagatca | 19 |
| **Ribosomal protein, large, P0 (RPLP0)**  ENSG00000089157 | ENST00000228306  ENST00000313104 | RPLP0CM0522F  cactgagatcagggacatgttg | RPLP0CM0598R  cttcacatggggcaatgg | 74 |
| **Target Genes** |  |  |  |  |
| **Citrate synthase (CS)**  ENSG00000062485 | ENST00000351328 | CSCM0319F  gcatcttgtcttgttcttgcag | CSCM0391R  ggtcagccaatatgtctttcaa | 69 |
| **Fatty acid synthase (FASN)**  ENSG00000169710 | ENST00000306749  ENST00000374646 | FASNCM0328UF  caggcacacacgatggac | FASNCM0419UR  cggagtgaatctgggttgat | 11 |
| **Fatty acid desaturase 3 (FADS3)**  ENSG00000149485 | ENST00000278829 | FADS3CM1423UF  ctggtggacatcgtcaggt | FADS3CM1499UR  tgttgccttcactgatggag | 20 |
| **Carnitine palmitoyltransferase 1A, liver (CPT1A)**  ENSG00000110090 | ENST00000265641(long) | CPT1AcomCM2371F  agttctcttgccctgagacg | CPT1AlongCM2519R  catcagaagagctcgttttcc | 3 |
| **Carnitine palmitoyltransferase 1B, muscle (CPT1B)**  ENSG00000100288 | ENST00000360719 | CPT1BcomCM2136F  gagcagcaccccaatcac | CTP1BcomCM2204R  aactccatagccatcatctgct | 10 |
| **CPT2 carnitine palmitoyltransferase II (CPT2)**  ENSG00000157184 | ENST00000287862  ENST00000371486 | CPT2CM0637F  ccaccatgcactaccagga | CPT2CM0696R  tggtgtcttcaagtttgggaat | 64 |
| **CD36**  ENSG00000135218 | ENST00000309881 | CD36CM0100F  gcatctgctcctgcaagact | CD36CM0199R  aattcgtctaatcattggaaagcta | 36 |
| **Acetyl-Coenzyme A carboxylase alpha (ACACA)**  ENSG00000132142 | ENST00000335166  ENST00000353139  ENST00000360679  ENST00000361253 | ACACAcomCM1138F  gatgtggatgatgggctaca | ACACAcomCM1200R  tgaggccttgatcattactgg | 73 |
| **Acetyl-Coenzyme A carboxylase beta (ACACB)**  ENSG00000076555 | ENST00000338432  ENST00000377848  ENST00000377854  ENST00000390027  ENST00000390028 | ACACBcomCM2231F  cttctcctggggagagaacc | ACACBcomCM2302R  cggatggacagttccttca | 7 |
| **Mitochondrially encoded cytochrome c oxidase I**  **(COX1 or MT-CO1)**  ENSG00000198804 | ENST00000361624  (no introns) | COX1CM0975F  cacactccacggaagcaata | COX1CM1049R  tcggtgaaaagaaagatgaatc | 84 |
| **Hydroxyacyl-Coenzyme A dehydrogenase,**  **alpha subunit (HADHA)**  ENSG00000084754 | ENST00000288682  ENST00000380649 | HADHAcomCM1978F  catggatagtattttagcgagtctga | HADHAcomCM2051R  ggaactggatgtcttcgtctg | 16 |
| **Hydroxyacyl-Coenzyme A dehydrogenase,**  **beta subunit (HADHB)**  ENSG00000138029 | ENST00000317799  ENST00000317815 | HADHBcomCM1380F  cactggctgcaggttggt | HADHBcomCM1498R  aagcttccactatcatagcatgg | 66 |
| **mtDNA to nDNA ratio** |  |  |  |  |
| **Mitochondrially encoded tRNA leucine 1 (UUA/G)**  **(MT-TL1)** – forward primer only  ENSG00000209082  **mitochondrially encoded NADH dehydrogenase 1**  **(MT-ND1)** – reverse primer only  ENSG00000198888 | N/A | mtDNATRNL1CM3246F  cccggtaatcgcataaaactt | mtDNAND1CM3389R  gaatttttcgttcggtaagca | 35 |
| **Adrenergic, beta-2-, receptor, surface (ADRB2)**  ENSG00000169252 | N/A | nDNACM0482F  gcctcttcctaaaatgcatca | nDNACM0541R  ctcacctcatatttgtgttttcca | 76 |
